# Small-scale sequencing enables quality assessment of Ribo-Seq data: an example from Arabidopsis cell culture

**DOI:** 10.1186/s13007-021-00791-w

**Published:** 2021-08-24

**Authors:** Amir Mahboubi, Nicolas Delhomme, Sara Häggström, Johannes Hanson

**Affiliations:** 1 Department of Plant Physiology, Umeå Plant Science Centre, Umeå University, Umeå, Sweden; 2 Department of Forest Genetics and Plant Physiology, Umeå Plant Science Centre, Swedish University of Agricultural Sciences, Umeå, Sweden

**Keywords:** Ribosomal profiling, Translation, Evaluation of sequencing library quality, Translational profiling, Ribo-Seq

## Abstract

**Background:**

Translation is a tightly regulated process, controlling the rate of protein synthesis in cells. Ribosome sequencing (Ribo-Seq) is a recently developed tool for studying actively translated mRNA and can thus directly address translational regulation. Ribo-Seq libraries need to be sequenced to a great depth due to high contamination by rRNA and other contaminating nucleic acid fragments. Deep sequencing is expensive, and it generates large volumes of data, making data analysis complicated and time consuming.

**Methods and results:**

Here we developed a platform for Ribo-Seq library construction and data analysis to enable rapid quality assessment of Ribo-Seq libraries with the help of a small-scale sequencer. Our data show that several qualitative features of a Ribo-Seq library, such as read length distribution, P-site distribution, reading frame and triplet periodicity, can be effectively evaluated using only the data generated by a benchtop sequencer with a very limited number of reads.

**Conclusion:**

Our pipeline enables rapid evaluation of Ribo-Seq libraries, opening up possibilities for optimization of Ribo-Seq library construction from difficult samples, and leading to better decision making prior to more costly deep sequencing.

**Supplementary Information:**

The online version contains supplementary material available at 10.1186/s13007-021-00791-w.

## Background

Translational regulation plays a prominent role in the expression of protein-coding genes, hence investigating gene expression based solely on transcriptome data may be insufficient [1–3]. With all the advances in sequencing technologies over the last decade, several experimental approaches have been developed to capture a global view of translation within a new branch of omics termed translatomics [4, 5]. Amongst the techniques for translatomics studies, Ribo-Seq is by far the most powerful of the methods that have been widely used over the recent years [6, 7]. This approach is based on the ability of ribosomes to protect 28–30 nt regions of the mRNA they enclose against nuclease digestion [8]. This feature of ribosomes, combined with state-of-the-art sequencing technologies, has made it possible to investigate translation in a quantitative and positional manner. Ribo-Seq has been successfully used with yeast and mammalian cells, leading to major breakthroughs in understanding the mechanisms behind translational regulation and discovering novel protein-coding regions of the genome [7, 9]. Examples in plants include investigating, for example, hypoxic stress [10], ethylene signaling [11], etiolation light/dark responses [12], and novel open reading frames (ORFs) discovery [13] in Arabidopsis.

A Ribo-Seq procedure consists of experimental and computational phases. The experimental phase comprises preparation of ribosome protected fragments (RPFs) by nuclease treatment, library construction, and sequencing; these are highly labor- and cost-intensive. The computational part involves preprocessing of Ribo-Seq reads followed by mapping them to a reference of choice which can be either a genome or a transcriptome. Mapping against a transcriptome reference is better suited to studies of translational efficiency and differential translation, whereas mapping against a genome reference is a standard procedure for discovering novel ORFs and association of ribosomes with non-protein-coding RNA. The binary alignment map (BAM) files resulting from mapping Ribo-Seq reads to the reference of interest can then be used for downstream P-site and triplet periodicity analyses, mainly using available R packages such as riboSeqR [14], riboWaltz [15] and RiboTaper [16].

Despite the use of biotinylated probes for negative subtraction of rRNA during library preparation, Ribo-Seq libraries still remain highly contaminated with ribosome-protected rRNA and other nuclease-resistant fragments [17]. This high proportion of rRNA in Ribo-Seq libraries necessitates increased sequencing depth in order to obtain a sufficient number of RPFs. Ribo-Seq experiments may therefore benefit from a suitable method for rapid quality assessment of libraries prior to more costly deep sequencing. The emergence of small-scale benchtop sequencers in recent years offers the possibility of quality assessing complex libraries like Ribo-Seq libraries in a time- and cost-efficient manner. Libraries that pass such a quality check (QC) can then be sent for deep sequencing, the estimates of rRNA contamination and required sequencing depth can potentially help to reduce the sequencing costs.

In this study, we compared libraries made from Arabidopsis dark-grown cells sequenced by small-scale and deep sequencing. The data were analyzed using our in-house Ribo-Seq data analysis pipeline and the results show that shallow sequencing can be used for quality assessment of Ribo-Seq libraries prior to deep sequencing. Various features indicating the quality of Ribo-Seq data, such as read length distribution, rRNA contamination, CDS enrichment, and triplet periodicity can be effectively evaluated from the data obtained by shallow sequencing; these are useful for optimization of library preparation protocols. Our Ribo-Seq pipeline provides a guideline for constructing high-quality Ribo-Seq libraries and a rapid way of quality assessing them with the help of small-scale sequencing.

## Methods

### Plant material and growth conditions

For T-0 samples, three biological replicates of dark grown Arabidopsis cells were cultured in 50 ml of growth medium (1 × MS + 3% sucrose) for 6 days. The T-3 samples were prepared by adding one volume of fresh growth medium to three biological replicates of T-0 samples followed by growing them for 3 h in the dark. Samples were harvested at 4 °C by passing the cultures through a filter paper under vacuum followed by quickly freezing the cells in liquid nitrogen. Cells were then ground with a mortar and pestle in liquid nitrogen and the powder was used for sample preparation.

### Preparation of RPFs

RPFs were obtained from the cell powders according to [13] by adding 400 µl of extraction buffer (Tris–Cl pH 8 100 mM, KCl 40 mM, MgCl2 20 mM, 2% Polyoxyethylene (10) tridecyl ether, 1% deoxycholic acid, 1 mM DTT, 100 ug/ml cycloheximide, DNase I 10 U/ml) to about 100 to 150 µl of cell powder on ice, incubating for 15 min on ice, and centrifuging for 15 min at 16,000×*g* at 4 °C. The supernatants were collected, and the RNA concentration was measured using a Qubit RNA broad range assay kit (ThermoFisher Scientific). The cell lysate samples were then digested with 30 units of Ambion RNase I (ThermoFisher Scientific) per 20 µg of RNA for 2 h at RT, then the digestion was stopped by adding 10 µl of SUPERase•In RNase inhibitor (ThermoFisher Scientific). The RPFs were purified from the digested samples according to the TRIzol reagent manual using 750 µl TRIzol (ThermoFisher Scientific), 150 µl chloroform, and overnight precipitation was carried out in 500 µl isopropanol and 5 µl glycogen (5 mg/ml ThermoFisher) at − 70 °C.

### Ribo-Seq library preparation

Library preparation was performed as described by [6] using about 20 µg of RNA. Briefly, the digested RNA samples were run through a 12 well 15% polyacrylamide gel with Tris–borate-EDTA (TBE) urea buffer (ThermoFisher Scientific) for 70 min at 200 V and RPFs between 27–31 nt were cut and extracted from the gel (Additional file 1: Figure S1). Gel extraction was performed by adding 400 µl of gel extraction buffer (300 mM sodium acetate pH 5.5, 1.0 mM EDTA and 0.25% SDS) to the excised gel pieces followed by gentle overnight rotation. Then 500 µl of isopropanol and 5 µl of glycogen were added to precipitate the RPFs followed by centrifuging at 20,000×*g* at 4 °C for 30 min to pellet the RPFs. The RPF pellets were subsequently resuspended in Rnase free water and used for library construction. T4 Polynucleotide Kinase (T4 PNK) (ThermoFisher Scientific) was then used to dephosphorylate the 3’ ends of the RPFs. T4 RNA Ligase 2, truncated (New England BioLabs) was used to ligate the Universal miRNA Cloning Linker (5′ rAppCTGTAGGCACCATCAAT–NH2 3′) (New England BioLabs) to the 3’ ends of the RPFs. The linker ligated samples were run through a 12 well 15% TBE urea gel, cut from the gel and extracted from the gel as described above. The cDNA was synthesized from the ligated RPF templates using Superscript III reverse transcriptase (ThermoFisher Scientific). The RNA was then eliminated from the cDNA synthesis reactions by adding 2.2 μl of 1 N NaOH to each reaction and incubating for 10 min at 80 °C followed by running through a 12 well 15% TBE urea gel and extraction from the gel as described above. In the next step, cDNA fragments were circularized using CircLigase ssDNA Ligase (Lucigen). The circularized cDNA fragments were then depleted of rRNA using biotinylated rRNA oligo probes shown in Additional file 1: Table S1 as described by [6]. The rRNA depleted libraries were finally amplified for 9 cycles using Phusion High-Fidelity DNA Polymerase (New England BioLabs) with the index primers shown in Additional file 1: Table S5. The amplified libraries were run through a 12 well 10% TBE gel (ThermoFisher Scientific) at 180 V for 70 min and gel extracted as described above. Purified libraries were then quantified using a NEBNext Library Quant kit according to manufacturer’s protocol (New England BioLabs).

### Sequencing and data preprocessing

About 50 pM and 1 nM libraries were multiplexed and used for iSeq100 and NovaSeq6000 sequencing respectively in 50 cycles and single end mode. The data has been deposited at the European Nucleotide Archive (ENA, https://www.ebi.ac.uk/ena/browser/home) under accession number PRJEB43647. The complete source code to reproduce the analysis is available from the GitHub repository https://github.com/nicolasDelhomme/riboSeqPipeline [https://doi.org/10.5281/zenodo.4603118]. Briefly, the FASTQ files from the sequencers were processed through our preprocessing pipeline by first quality checking using FastQC (v0.11.4, http://www.bioinformatics.babraham.ac.uk/projects/fastqc/), followed by filtering the rRNA using SortMeRNA (settings –log –paired_in –fastx–sam –num_alignments 1) (v2.1b, [18] against rRNA databases from the small and large subunits of the archaea, bacteria and eukaryotes (Additional file 1: Table S2), supplemented with *Arabidopsis thaliana* rRNA sequences (Additional file 1: Table S3) before removing the sequencing adapters and quality trimming the reads using trimmomatic (settings NEB-universal-adapter.fa:1:15:10 SLIDINGWINDOW:5:20 MINLENGTH:16) (v0.39, [19]. After both filtering steps, FastQC was run again to ensure that no technical artefacts were introduced. The rRNA-filtered and adapter-cleaved fastq files were then used for mapping and quantification, applying bowtie2 (v2.3.5.1 with default parameters, [20] and kallisto (v0.44 with default parameters, but run against a database generated with a kmer size of 15, [21] on the ARAPORT11 genome and transcriptome references, respectively. MultiQC [22] was used to generate a summary report from the overall preprocessing procedure.

### Ribo-Seq data analysis

The source code to reproduce all analyses is available from the GitHub repository https://github.com/nicolasDelhomme/riboSeqPipeline [https://doi.org/10.5281/zenodo.4603118]. All the Ribo-Seq data analysis was performed in R (v4.0.3, [23] using the packages riboWaltz (v1.1.0, [15] and GenomicFeatures (v1.26.7, [24] for read length distribution plot, P-site and periodicity calculation, and generation of the heatmap plots. Spearman correlation analysis was performed using the ggpubr package (v0.4.0) in R and the regression lines were locally corrected for the best fit. The Arabidopsis genome annotation file (Araport11_GFF3_genes_transposons.201606.gtf) retrieved from arabidopsis.org was used as the annotation file in this analysis. Statistical analysis of transcript differential expression between conditions was performed in R using the Bioconductor DESeq2 package (v1.28.0, [25] with an adjusted p-value cutoff of 0.05 for significance assessment. The Venn diagram was made using the LIMMA package (v3.44.1, [26] in R.

## Results

### Preprocessing and assessment of Ribo-Seq libraries generated from Arabidopsis cell culture

**Fig. 1:**
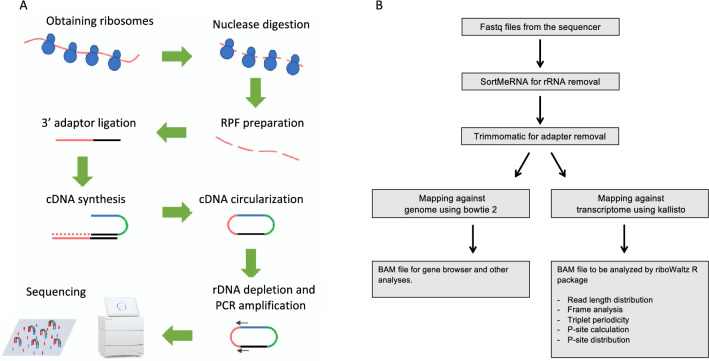
**A** Overview of Ribo-Seq library construction pipeline from RPF preparation to sequencing. **B** Flowchart representing the Ribo-Seq data analysis pipeline developed for this study

**Fig. 2:**
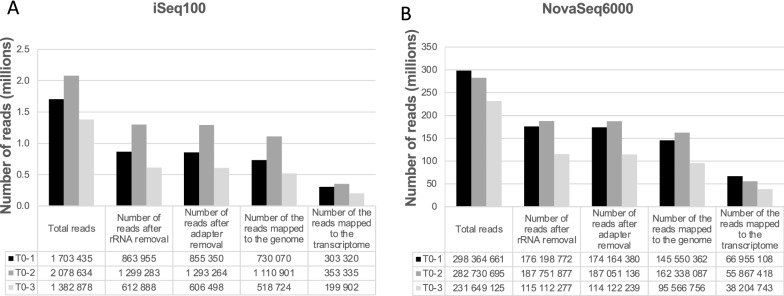
Overall quantitative representation of Ribo-Seq data analyzed by MultiQC, showing the read numbers at each step of the preprocessing for the iSeq100 (**A**) and NovaSeq6000 (**B**) data

We generated Ribo-Seq libraries from three biological replicates of 6-day old Arabidopsis cell culture (T0-1 to T0-3) using the pipeline illustrated in Fig. 1A. The RPFs were generated from crude cellular extract that was previously shown to be robust and accurate [13, 27]. The libraries were depleted from rRNA using biotinylated probes designed against the most abundant rRNA fragments (Additional file 1: Table S1). The libraries were then sequenced using an Illumina iSeq100 benchtop sequencer, for small-scale sequencing, and an Illumina NovaSeq6000, for deep sequencing. The reads from both sequencers were subsequently preprocessed, mapped, and analyzed using our in-house Ribo-Seq data analysis pipeline, which is depicted in Fig. 1B. For the iSeq100 data, the initial number of reads for the three biological replicates ranged from approximately 1.4 to about 2.0 million reads, which reduced by 38 to 56 percent after rRNA filtering and 3’ adapter removal (Fig. 2A). NovaSeq6000 generated approximately 230 to 300 million reads for each of the three biological replicates, reducing by about 35 to 50 percent after rRNA filtering and 3’ adapter removal (Fig. 2B). Almost all the reduction in the number of reads was due to the removal of contaminating rRNA and it was in about the same range across both instruments. SortMeRNA [18] was used to remove rRNA, it uses a handful of built-in eukaryotic and prokaryotic rRNA databases as reference sequences for filtering. The sequences of Arabidopsis 5S, 5.8S, 18S, and 25S rRNA genes were also included as an additional SortMeRNA database and shown to improve the rRNA filtering efficiency. The rRNA composition in the data generated by both iSeq100 and NovaSeq6000 was fairly similar, as 18S and 25S represented the majority of the filtered rRNA with some 23S rRNA present, which is most likely of plastidial or mitochondrial origin (Additional file 1: Figure S2A and B).

The reads were aligned to the Arabidopsis transcriptome and genome using kallisto and bowtie 2 respectively. Mapping to a transcriptome reference allows focusing solely on known genes, mostly protein-coding. The reason for choosing kallisto over bowtie 2 for transcriptome mapping is that kallisto is faster and more accurate than bowtie 2 [21] (Additional file 1: Table S4). The number of reads after mapping clearly show that proportionally fewer reads were mapped to the transcriptome than to the genome for both iSeq100 and NovaSeq6000 data (Fig. 2). Integrated genome browser [28] snapshots taken from a randomly selected region on chromosome 1 using the genome mapped data from both sequencers show that the majority of the reads were mapped to exonic regions of the genome with some putatively translated regions not listed in the annotation (Additional file 1: Figure S3).

**Fig. 3:**
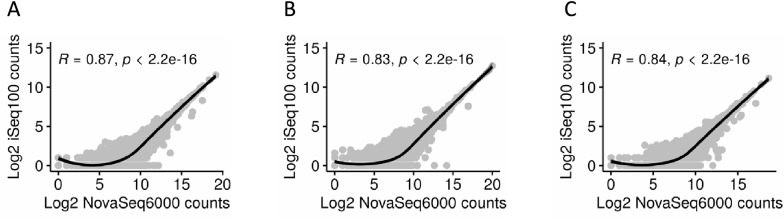
Scatter plot showing NovaSeq6000 versus iSeq100 log_2_ of read counts per transcript for T0-1 (**A**), T0-2 (**B**), and T0-3 (**C**) samples. Spearman correlation coefficients (R) and *p-*values are indicated above the plot. The regression lines were locally corrected for the best fit

We also performed Spearman’s correlation analysis on T-0 transcriptome mapped data to find out if there is a correlation between the iSeq100 and NovaSeq6000 read counts per transcript. High degree of correlation was observed between the iSeq100 and NovaSeq6000 log_2_ of read counts per transcript in all three biological replicates as was evident from the correlation coefficient values (R) and the *p*-values presented (Fig. 3).

### The key Ribo-Seq quality assessment criteria are nearly identical for iSeq100 and NovaSeq6000 datasets

**Fig. 4:**
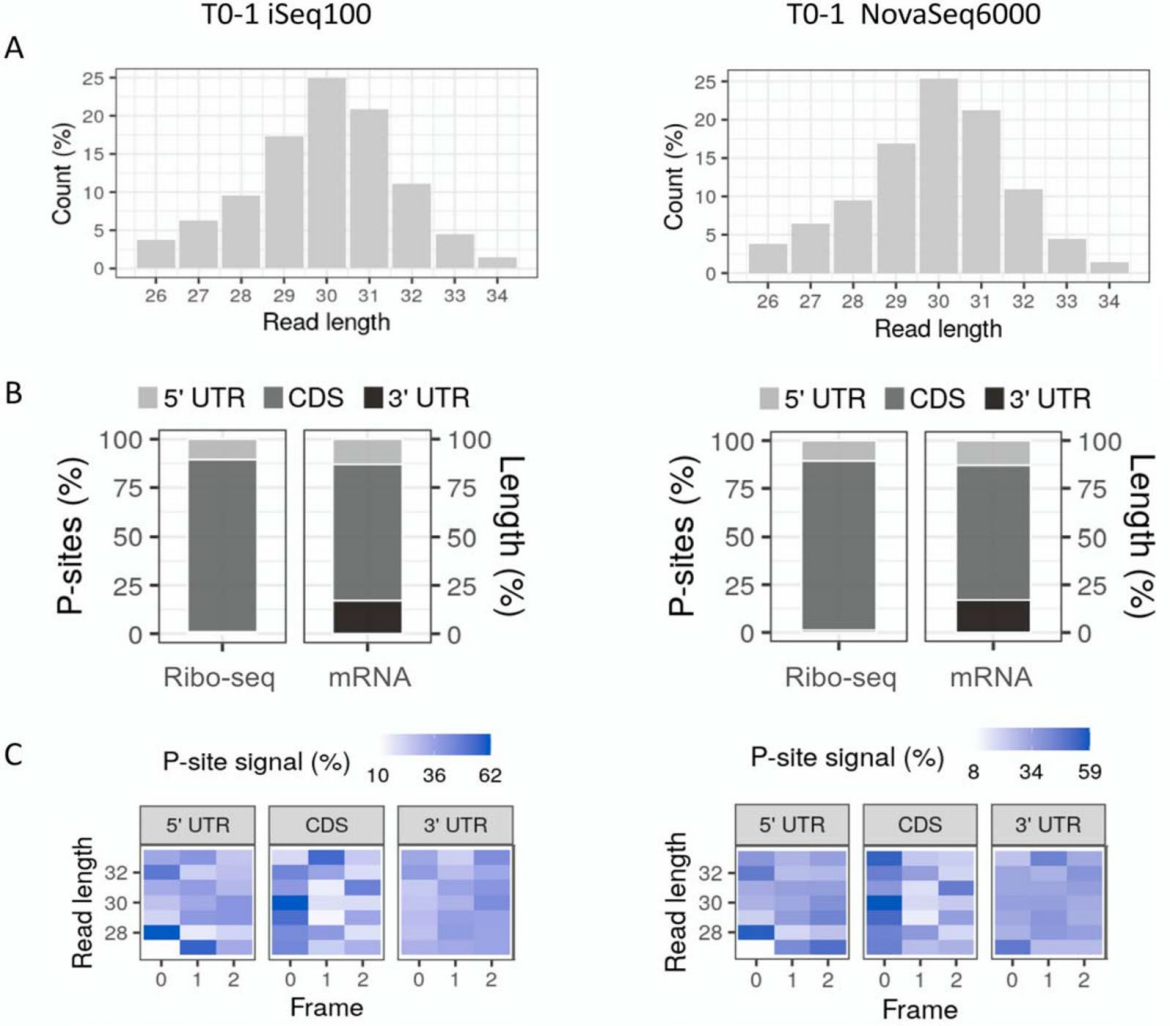
Read length distribution (**A**), percentage of P-site distribution along the 5’ UTR, CDS and 3’ UTR of the mRNA sequences in the Araport11 annotation file (right) and Ribo-Seq data (left) (**B**) and read length specified percentage of P-sites in three frames within the 5’ UTR, CDS, and 3’ UTR regions of the transcripts. **C** For the T0-1 data generated by iSeq100 and NovaSeq6000

**Fig. 5:**
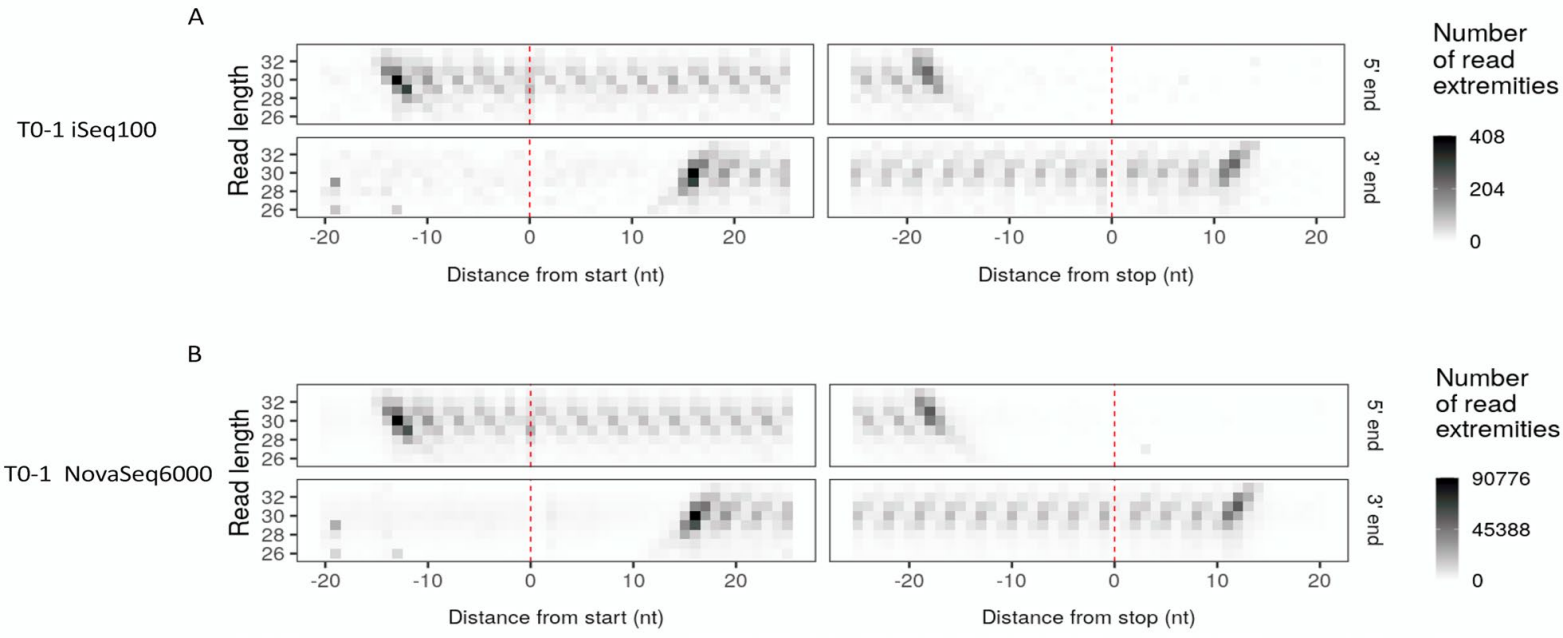
Meta-gene heatmap illustrating the frequency of the reads at the 5’ and 3’ ends, around the start and stop codon for different read lengths in the T0-1 data generated by iSeq100 (**A**) and NovaSeq6000 (**B**)

Results from transcriptome mapping were used to calculate the key quality criteria for Ribo-Seq data: read length distribution, P-site distribution within the transcripts, triplet periodicity, and read distribution around the translation start and stop. Read length distributions of iSeq100 and NovaSeq6000 data were identical, spanning from about 26 nt to 34 nt with a peak at 30 nt (Fig. 4A, Additional file 1: Figure S4A). The P-site distribution along the 5’UTR, CDS and 3’UTR of the transcripts were also calculated and compared to the distribution of the three regions in all the mRNA sequences in the transcriptome annotation file. Results showed higher P-site enrichment in the CDS region of the transcripts for the Ribo-Seq data compared to CDS proportion from the Arabidopsis mRNA sequences, as expected (Fig. 4B, Additional file 1: Figure S4B). Slightly fewer and nearly no P-sites were associated with the 5’UTR and 3’UTR of the Ribo-Seq data respectively. The pattern of P-site distribution showed a high degree of similarity between iSeq100 and NovaSeq6000 data. We also performed frame analysis of the P-sites mapped to the three frames of the mRNA sequences. P-sites calculated from the reads were randomly distributed in all the frames for different read lengths at the 3’UTR of transcripts, whereas frame bias was evident mainly for 28 nt and 32 nt reads at the 5’UTR as well as all the reads, especially 30 nt reads, in the CDS regions of transcripts (Fig. 4C, Additional file 1: Figure S4C). This bias towards frame 0 was seen in both iSeq100 and, to a slightly greater extent, NovaSeq6000 data. We then created a metagene heatmap showing the read extremities at translational start and stop sites for the data generated by both sequencers. As depicted in Fig. 5 (also in Additional file 1: Figure S5) for both sequencers, RPFs ranged from 28 to 32 nt, with the 5’ ends starting and peaking at 12 and 13 nt upstream of the translational start site for 29 and 30 nt reads respectively and showed clear periodicity especially in the case of 30 nt reads. The RPFs 5’ extremity showed a continuous periodic pattern that ended 18 nt upstream of the stop codons for both 29 and 30 nt reads. The 5’UTR and particularly 3’UTR regions showed nearly no RPF association. The heatmaps of the data produced from iSeq100 and NovaSeq6000 are very similar, with visibly reduced background signals particularly within the 5’UTR region in the NovaSeq6000 metagene heatmap.

### Small-scale sequencing data can be used for preliminary evaluation of the Ribo-Seq data

**Fig. 6:**
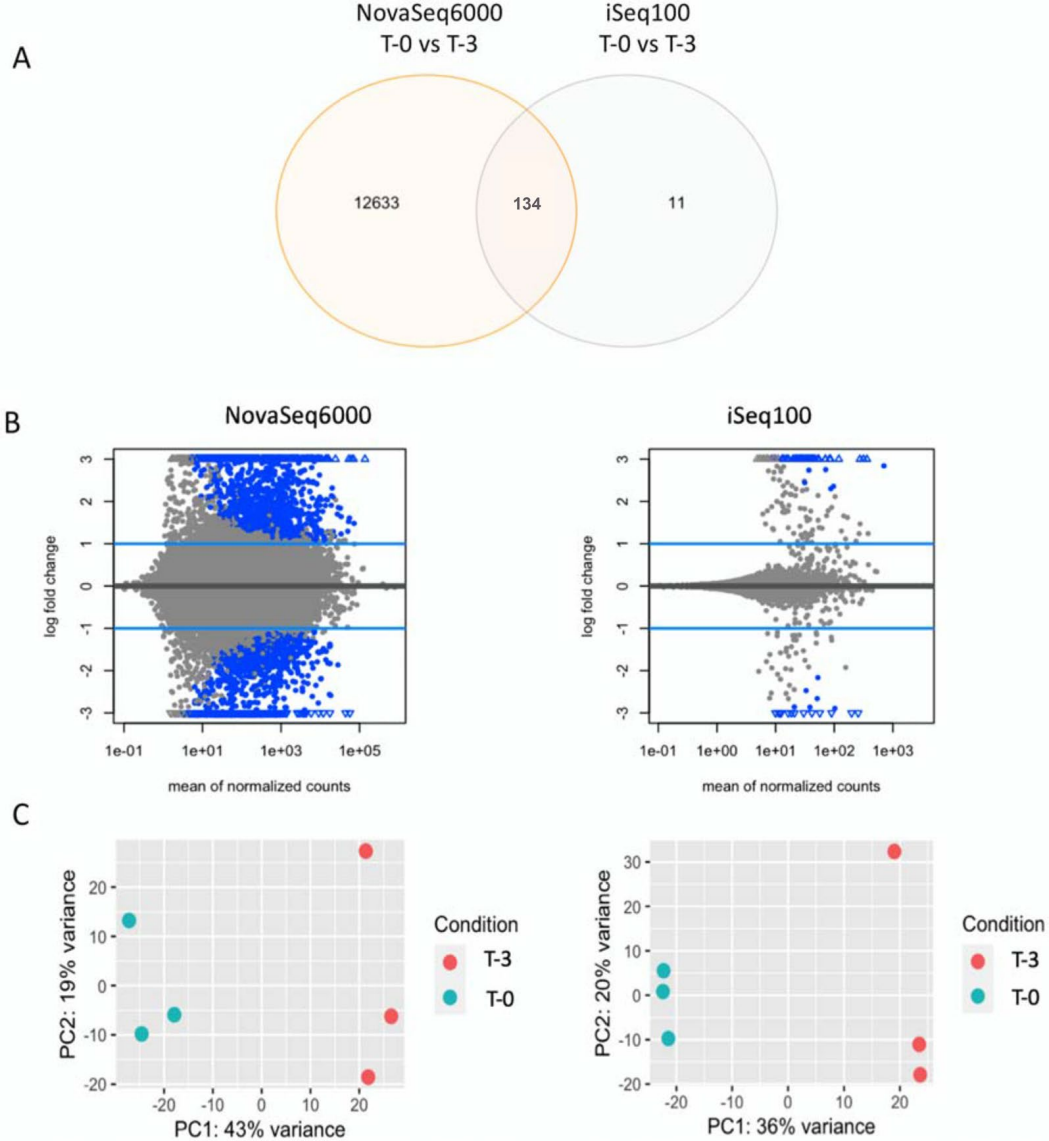
**A** Venn diagram representing the number of differentially expressed genes with adjusted p-values less than 0.05 for iSeq100 and NovaSeq6000 data. **B** MA-plot showing the log2 ratio fold change vs the mean of normalized counts for the data generated by iSeq100 and NovaSeq6000; the blue points have adjusted p-values less than 0.1. **C** Principal component analysis, showing the first two components causing separation between T-0 and T-3 samples for the data generated by iSeq100 and NovaSeq6000. The comparisons were made using three biological replicates of each treatment

To assess the predictive power of the iSeq100 small-scale sequencer on Ribo-Seq data, we performed differential expression analysis between the T-0 samples and the T-3 samples. DESeq2 [25] was used to obtain the differentially expressed genes (DEGs) between the two conditions, using three biological replicates per treatment. The number of DEGs for the two conditions were 12,767 and 145 for Novaseq6000 and iSeq100 datasets respectively, of which 134 DEGs were common between the two datasets (Fig. 6A). The MA-plots (plots showing the log2 ratio fold change vs the mean of normalized counts) showed a much greater number of differentially expressed genes (blue dots) in the data generated by NovaSeq6000 (Fig. 6B). Nonetheless, the principal component analysis plot (PCA) showed a similar grouping of the T-0 and T-3 samples for both iSeq100 and NovaSeq6000 data (Fig. 6C). The RPF profiles of the highly abundant transcripts showed very high similarities between the iSeq100 and NovaSeq6000 data; the profiles for the two most abundant transcripts are shown in Additional file 1: Figure S6.

### Small-scale sequencing saves cost and time for QC of Ribo-Seq libraries

We estimated the sequencing cost of our Ribo-Seq libraries as well as the time needed for sequencing and data analysis for both sequencers. Based on our estimates, the sequencing cost of iSeq100 per sample is 3.25 times lower than NovaSeq6000. Additionally, the time needed for sequencing (mainly shipment and queueing time), preprocessing and data analysis for the iSeq100 is remarkably shorter than what is needed for Novaseq6000. The data obtained from iSeq100 can be sequenced, fully processed and analyzed in less than a day per sample whereas the NovaSeq6000 generates data that need a few days per sample to be processed and analyzed. Moreover, the deep sequencing is often performed off-site which can result in longer total processing times (Table 1).

**Table 1:** Comparison of estimated sequencing cost and data processing time per sample between iSeq100 and NovaSeq6000

	iSeq100	NovaSeq6000
Cost per sample (EUR)	200	650
Estimation of analysis time per sample (hours)	1	96
Estimation of shipment and queue time (days)	1	30
Estimation of read per sample (million)	1.5	200

## Discussion

High levels of contamination with rRNA, tRNA and other ribosome-protected RNA fragments remain the main challenge in reducing the cost of sequencing Ribo-Seq libraries while still being able to generate a sufficient number of RPFs for statistically sound analyses. In this study, rRNA comprised approximately 35 to 56 percent of the library contents and was removed during the data preprocessing. The rRNA content of the libraries was substantially lower than the rRNA percentage expected for Ribo-Seq libraries without rRNA depletion, which can reach up to 90 percent of the total reads [17]. This shows that our use of biotinylated rRNA oligo probes for rRNA removal during library construction removed around 30 percent of the rRNA. Currently, we are using the shallow sequencing data for designing additional biotinylated probes that can be used to remove other overrepresented contaminating fragments, in particular tRNAs, from our libraries.

In our data analysis pipeline, we implemented the option to map the reads to an Arabidopsis transcriptome reference. Although outputs from data that were aligned to transcriptome references are likely to lack information on unannotated ORFs, their smaller size is well suited for differential translation studies and calculations of translational efficiency. Transcriptome mapping was also used as a part of the Ribo-Seq data QC for faster and more efficient processing in this study. Our data from both sequencers show about 70 percent reduction in the number of reads that were aligned to the transcriptome compared to the genome, providing us with enrichment for protein coding regions of the genome and a more manageable output files for further downstream processing. The majority of reads that were not aligned to the transcriptome most likely come from unannotated ORFs and non-coding regions of the genome such as the remaining rRNA (after SortMeRNA filtering), tRNA and small nucleolar RNA that also resist the nuclease digestion [13, 29].

In addition to the high correlation between the read counts per transcript in logarithmic scale, iSeq100 and NovaSeq6000 datasets were also shown to be highly similar in several key Ribo-Seq QC parameters. The read length distribution allows us to accurately evaluate the span of the read lengths and where they peak based on the small-scale sequencing data and to check whether they follow the pattern normally expected for Ribo-Seq data [13]. The peak observed at 30 nt is within the normal peak range expected for Ribo-Seq data (28 nt to 30 nt), this can vary depending on the nuclease concentration used for the digestion, duration of digestion, and also ribosome conformation at the site of translation [30, 31]. RPFs are also expected to be more enriched in CDS-aligning fragments with less association of reads to the UTRs, especially the 3’UTR, as they are not normally translated [7]. The noticeable number of RPFs associated with the 5’UTR in the current study is likely due to the presence of known translational regulatory elements within the 5’UTR such as upstream ORFs (uORFs) and internal ribosome entry sites (IRES) [32].

Nuclease digestion of translating ribosomes, treated with cycloheximide, generates in-frame RPFs, thus the P-sites calculated for each RPF length are expected to have periodic patterns [30]. In the iSeq100 data, the clear frame 0 bias within the CDS was improved by increased sequencing depth as was clear from the NovaSeq6000 data. Nevertheless, it is still possible to make fair assessments of the translating frame based solely on the iSeq100 data. Less background observed in the metagene heatmap for the NovaSeq6000 data also suggests that increasing sequencing depth reduces background and results in sharper read extremities. However, the iSeq100 depth is powerful enough to give a clear image of the triplet periodicity and overall shape of the libraries. The UTRs, in particular 3’UTR, were devoid of RPFs and the 5’ ends of the reads started to peak at 12–13 nt, continuing to 18 nt upstream of the translational start and stop sites. These are the patterns that are normally expected for Ribo-Seq reads. The fact that such patterns can be rapidly obtained from the iSeq100 data provides an efficient way of Ribo-Seq QC and troubleshooting.

The comparisons between T-0 and T-3 treatments were made in order to see whether any differences and/or patterns can be observed in the small-scale sequencing data, potentially giving hints about general tendencies in the treatments. It is obvious that there was a huge difference between the two datasets in the number of DEGs as a result of limited depth delivered by iSeq100, but on the other hand the separation of samples belonging to each treatment was similar in the iSeq100 and the NovaSeq6000 data, as depicted in the PCA plot. Moreover, the majority (134 out of 145) of DEGs from the iSeq100 dataset were also found in the NovaSeq6000 dataset, justifying the grouping of the samples in the PCA plot. Consistently, similar RPF profiles of the highly abundant transcripts in both sequencing datasets also support the fact that the iSeq100 data is a small-scale representation of the NovaSeq6000 data. There are tools that can be used to predict the optimal depth for Ribo-Seq library sequencing based on shallow sequencing data, such as superSeq [33] and RiboSimR [34]. However, they are very unlikely to give accurate estimates of the optimal depth since there are orders of magnitude difference in sequencing depth between the iSeq100 and NovaSeq6000 outputs, making the predictions fall outside the standard range.

## Conclusion

In this study, we present a method for quality assessment of Ribo-Seq libraries by shallow sequencing using Arabidopsis cell culture material as an example. Key quality features of a Ribo-Seq data can be easily evaluated using merely the reads obtained by shallow sequencing. Our method can possibly allow preliminary identification of differences between the experimental treatments. We suggest that the method can be applied to other organisms contingent on sufficient removal of contaminating fragments from the libraries both through biochemical depletion and bioinformatic filtering. Our results show that Ribo-Seq libraries can be quality assessed with high precision by using only about 1.5 million reads, and this can save time and costs allowing better decision making before progressing to the actual deep sequencing.

## Supplementary Information

**Additional file 1: **Table S1. Biotinylated rRNA depletion probes. Table S2. List of rRNA databases used with SortMeRNA for rRNA removal. Table S3. Arabidopsis rRNA fasta file added to the SortMeRNA database. Table S4. Mapping quality comparison between bowtie 2 and kallisto. Table S5. Library amplification primers. Figure S1. Gel images of the library construction procedure. Figure S2. The rRNA compositions of the libraries. Figure S3. Genome browser (IGB) view of a randomly selected region. Figure S4. Ribo-Seq QC analysis of T0-2 and T0-3 data. Figure S5. Meta-gene heatmap of T0-2 and T0-3 data. Figure S6. The RPF profiles of the two most abundant transcripts.

## Data Availability

Raw data has been deposited at the European Nucleotide Archive (ENA, https://www.ebi.ac.uk/ena/browser/home) under accession number PRJEB43647. The complete source code to reproduce the analysis is available from the GitHub repository https://github.com/nicolasDelhomme/riboSeqPipeline [https://doi.org/10.5281/zenodo.4603118].
